# The American Illustrated Medical Dictionary

**Published:** 1900-12

**Authors:** 


THE AMERICAN ILLUSTRATED MEDICAL DICTIONARY. A New and Complete Dictionary of the Terms Used in Medicine, Surgery, Dentistry, Pharmacy, Chemistry and the Kindred Branches. With their Pronunciation, Derivation, and Definition. Including much Collateral Information of an Encyclopedic Character. By W A. NEWMAN DORLAND, A. M , M. D., Assistant Obstetrician to the University of Pennsylvania Hospital; Editor of the American Pocket Medical Dictionary. With numerous illustrations and 24 colored plates. Philadelphia and London: W. B. Saunders & Co. 1900. [Price, $4 50, plain; $5.00, index ]

One of the beauties of this book is that it is easy and comfortable to handle with its flexible covers; easy to read with its remarkably clear type and its good quality of paper. For these benefits we must extend thanks to the publishers, W. B. Saunders & Co. They have put forth a work which is up to the Saunders standard, which appears always to be to give the best in its most convenient and usable form. One would not expect Saunders to hand out a hard-shell dictionary; neither would one expect to see come from their presses a book, say on practical analysis of urine—a sort of work-table guide— in pretty, soft covers, with uncut or deckel edges. There is always a fitness of things about Saunders's publications which inspire confidence.

As to Dr. Dorland's share in the work of producing this dictionary too much cannot be said in way of praise and congratulation. A careful and serious search,—comparative, really,—of the work fails to reveal anything within reason, which has been left undone in the way of producing a perfect dictionary. All the ordinary terms used in medicine, surgery, dentistry, pharmacy, chemistry and the kindred branches, with pronunciation, derivation and definition are given, and in a most assimilable form. In connection with all this is much information of an encyclopedic character, although there is not by any means any effort shown to masquerade the book as an encyclopedia. It is just a word book, convenient and useful and one who uses it as a guide and sets it up as a standard cannot go astray in his work. There is much matter which appears in tabular form, including anatomical and clinical material, tests, stains and staining methods, treatment, diseases, and the like. One of the distinct features of Dr. Dorland's work is the careful attention which has been devoted to pronunciation and derivation. He has used a system for expressing sounds which is so simple and yet so accurate that one instinctively wonders how in the world any medical man can persist in saying *ab*-domen and par-*e*-sis with such a book in easy reach. The book is splendidly illustrated,—not for the sake of using pretty pictures, but to aid in a better understanding of the text. Amputations are illustrated perfectly by outline figures, and under arteries, nerves and veins are sketches in appropriate and distinctive colors and descriptive tables. The various microörganisms of disease are shown in color; bandaging is illustrated and blood affections are most beautifully shown in exquisite plate work. Fractures, muscles and sutures and ligaments are shown in black and white, and embryology is made clearer by the use of colored plates.

Altogether Dr. Dorland's dictionary is just such a handy, companionable book as a doctor could use with great benefit to himself individually and to the profession in general, if he kept it within easy reach on his table.—N. W. W.

